# Disintegrating the Structure and Improving the Functionalities of Pea Fiber by Industry-Scale Microfluidizer System

**DOI:** 10.3390/foods11030418

**Published:** 2022-01-31

**Authors:** Xiaohong He, Taotao Dai, Jian Sun, Ruihong Liang, Wei Liu, Mingshun Chen, Jun Chen, Chengmei Liu

**Affiliations:** 1 State Key Laboratory of Food Science and Technology, Nanchang University, Nanchang 330047, China; hexiaohongmaimai@163.com (X.H.); liangruihong@ncu.edu.cn (R.L.); liuwei@ncu.edu.cn (W.L.); chenshun1221@163.com (M.C.); chen-jun1986@hotmail.com (J.C.); 2 Agro-Food Science and Technology Research Institute, Guangxi Academy of Agricultural Sciences, Nanning 530007, China; ncubamboo@163.com (T.D.); jiansun@gxaas.net (J.S.)

**Keywords:** pea fiber, industry-scale microfluidizer, structure, functional properties

## Abstract

In the food industry, the most prominent and concerned points in the application of dietary fiber are hydration properties and oil absorption capacity. The target of this work was to investigate the impact of a novel industry-scale microfluidizer system (ISMS) on the changing structures and functionalities of pea fiber. Different ISMS treatment intensity (0–120 MPa for one pass and 120 MPa for two passes) was applied to treat pea fiber. ISMS treatment induced the reduction in particle size and the transformation of big compact blocks to loose flakes, and the destruction of the original ordered cellulose structure caused the decline of crystallinity. Meanwhile, the hydration properties of pea fiber were improved, and pre-pulverizer and industry-scale microfluidizer treatment together increased the swelling capacity and water retention capacity of fiber. The oil holding capacity of ISMS-treated fiber was increased to more than double the original one. The elevated functionalities of pea fiber by ISMS treatment could be attributed to loosening structure, exposing more surface area, and disordering the crystalline structure, which increased the sites of water binding and oil adsorption. These findings suggested that ISMS could be applied as an effective industrial technique to the disintegrate structure and improve the functionalities of pea fiber, so as to widen the application of pea fibers in foods.

## 1. Introduction

The appropriate consumption of dietary fibre has considered to be advantageous to health, such as by preserving gastrointestinal function, lowering blood lipids and cholesterol levels, and reducing the risk of cardiovascular disease [1], so we were encouraged to seek excellent fiber sources for the development of foods supplemented with dietary fibers. Pea fiber is a valuable and attractive food component which has been added to pasta for improving the nutritional value [2]. Commonly, the incorporation of raw pea fiber would negatively impact the sensory characteristics of the food matrix owing to its poor techno-functional properties. One of the areas of focus among researchers and in the field of functional food processing is the modification of fibers to increase their quality or functional properties by adopting physical, chemical and biological approaches [3]. In recent years, superfine pulverization techniques have gained much attention for their modifying properties of fiber. For instance, pulverization methods such as homogenization, microfluidization and ultrafine comminution have been reported to modify fibers from citrus [4,5], purple-fleshed potatoes [6], bamboo shoot shells [7] and carrot pomace [8]. Furthermore, microfluidization showed great potential in modifying fibers such as wheat and corn bran, peach and oat fiber, insoluble soybean fiber and hazelnut skin fiber, as reviewed by Guo, et al. [9] and Ozturk and Turasan [10]. Morales-Medina, et al. [11] also found a continuous defibrillation of pea hull fiber by microfluidization with the decreasing of particle size. Nevertheless, previous reports were mainly concentrated on treating fibers by small scale microfluidizers. As pointed out by Ozturk and Turasan [10], one of the biggest problems with this technology was the limitation of scale, and additional supporting equipment like a pretreatment miller was necessary if an industry-scale microfluidizer was provided. In our research group, an innovative industry-scale microfluidizer system (ISMS) was developed which combined a pre-pulverizer and an industry-scale microfluidizer (ISM). The schematic diagram and the main functions of the pre-pulverizer and industry-scale microfluidizer were demonstrated in our previous research in greater detail [12,13,14]. The industry-scale microfluidizer possesses a unique constructional microchannel with large orifice diameters, unusual impact modes and a high processing capacity which could continuously run to reach productivity of five tons per hour. ISMS has been successfully applied to produce stable whole soybean milk and improve the stability of whole corn slurry without filtering and removing any components. This indirectly reflected that the soybean and corn fibers in the whole component systems were modified. With regard to the low utilization of pea fiber, it was worth investigating the effect of ISMS treatment on its structural and functional properties. Ascertaining the efficiency and ability of ISMS to modify pea fiber can provide the possibility for its high-value utilization.

Therefore, the objective of the current work was to investigate the efficiency of ISMS modifying pea fiber. Pea fiber was treated by ISMS at a different intensity (60, 90, 120 MPa for one pass and 120 MPa for two passes after pre-pulverizer treatment). Subsequently, the structure and functionalities of ISMS-treated pea fiber (ISMS-treated PeaF) were determined. The structural properties were characterized by particle size distribution, confocal laser scanning microscopy (CLSM), scanning electron microscopy (SEM), and X-ray diffraction (XRD) analysis. The functionalities including swelling capacity (SC), water retention capacity (WRC) and oil holding capacity (OHC) of ISMS-treated PeaF were discussed. This study may identify a new technology that can be utilized to manufacture nutritious foods with high fiber content, thereby promoting the development of new food products.

## 2. Materials and Methods

### 2.1. Material

Pea fiber powder was provided by Shuangta Food Co., Ltd (Zhaoyuan, China), which contained 80.7% of total dietary fibre. The protein, starch, and ashes accounted for a much lower percentage (0.3, 3.6 and 3.4%, respectively). Calcofluor white was purchased from Aladdin Reagent Company (Shanghai, China). Corn oil was purchased from a local supermarket (Jinlongyu, Shanghai, China). All other chemical agents were of analytical grade.

### 2.2. ISMS Treatment of Pea Fiber

Native pea fiber (native PeaF) suspensions were prepared by mixing pea fiber powder in distilled water using a stirrer for overnight at approximately 500 rpm to completely hydrate, and a concentration of 1.2% (*w*/*w*) was selected according to the investigation of Bruno, et al. [5]. Completely hydrated pea fiber suspensions were added directly into the pre-pulverizer, then the pea fiber suspensions obtained from the pre-pulverizer were treated by ISM for one pass at 60, 90, and 120 MPa and two passes at 120 MPa in series. The temperature of the treated pea fiber suspensions (ISMS-treated PeaF) was less than 54 °C, and after ISM treatment, the ISMS-treated PeaF suspensions were immediately cooled to room temperature using an ice bath. Pea fiber treated by ISM at the corresponding intensity was designated as ISM-60 PeaF, ISM-90 PeaF, ISM-120 PeaF and ISM-120-T_2_ PeaF, respectively. The fiber treated by pre-pulverizer but without ISM treatment was labeled as Pre-PeaF. Part of the suspensions were withdrawn to perform particle size distributions and CLSM analysis. Other suspension samples were freeze-dried and ground into powders. The suspensions were first frozen at −80 °C overnight, and then freeze-dried using the FreeZone^®^ 4.5 L freeze-drier (Labconco, Kansas City, MO, USA) at low vacuum (≤10 bar) and temperature (~−40 °C). The freeze-dried PeaF was ground into power using a basic analytical mill (IKA^®^ A11B S025, Merck KGaA, Darmstadt, Germany) by intermittent operating for dozens of seconds. The obtained powders were stored in a desiccator for further analysis.

### 2.3. Particle Size

The particle size distributions and mean diameters of pea fibers were determined (expressed in % volume) using a Malvern MasterSizer 3000 (Malvern Instrument, Ltd., Malvern, UK) by referencing the method of Chen, et al. [15] with slight modifications. This technique provides information on the equivalent sphere diameter of fiber particles with different geometrical shapes, and the term “diameter” will be used to substitute for “equivalent sphere diameter” for simplicity. The measurement was conducted with the refractive indices of 1.52 and 1.33 for pea fiber and water, respectively, and an absorption index of 0.1 was used. The mean diameters including volume weighted mean diameter D_[4,3]_ (diameter of the sphere of equivalent volume to measured particles) and surface-weighted mean diameter D_[3,2]_ (particle diameter that has the same specific surface as that of the full distribution) were evaluated. Cumulative percentiles of D_10_, D_50_ and D_90_ were also calculated, which indicated that the size of 10%, 50% and 90% of the particles was below the specified diameter, respectively. The pan of particle size distributions was applied to characterize the width of the particle size distribution, which was calculated according to Equation (1):

(1) $$Span=\frac{D_{90}-D_{10}}{D_{50}}$$

### 2.4. Confocal Laser Scanning Microscopy (CLSM) Analysis

The microstructure of pea fiber was observed using confocal laser scanning microscopy (Carl Zeiss LSM710, Jena, Germany). The preparation of samples were conducted by referencing the method of Huang, et al. [16]. Briefly, pea fibers were mixed with calcofluor white dye at a 50:1 volume ratio, and then the stained sample was added to a culture dish for observation. Images were acquired using a CLSM multiphoton system with a 40× objective lens. The excitation-emission wavelengths of 405–455 nm for calcofluor white and appropriate emission channels were used.

### 2.5. Scanning Electron Microscopy (SEM) Analysis

The morphology of pea fiber samples after freeze-drying and grinding was also examined using an environmental scanning electron microscope (Quanta-200, FEI Company, Eindhoven, The Netherlands). ISM-treated PeaF were prepared by sticking them onto double-sided adhesive tape attached to a circular specimen stub, following by sputtering a thin film of gold. The morphology of samples was observed at ×800 magnifications with an accelerating voltage of 5 kV voltage.

### 2.6. Bulk Density Analysis

1.8 g of pea fiber powder was accurately weighed and carefully added into a calibrated 25 mL graduated cylinder. Pressure was imposed manually to ensure no further decrease in sample volume. The bulk density was calculated as the volume of sample occupied by per gram dry weight (mL/g) [17].

### 2.7. X-ray Diffraction (XRD) Analysis

X-ray diffractograms of pea fiber powders after freeze-drying and grinding were obtained using an X-ray diffractometer (D8 Advance, Bruker, Berlin, Germany) operated at 40 kV and 40 mA with Cu Kα radiation. Before measurements, samples were stored in a desiccator where a saturated solution of NaCl maintained a constant humidity atmosphere (relative humidity = 75%) at 25 °C for five days. The scanning angle (2θ) of 5–50° with the interval of 0.02° was used to obtain XRD patterns. The crystallinity of samples was calculated based on the ratio of the area of the crystalline region to the total area in the XRD spectra using Origin software [18].

### 2.8. Measurement of Hydration Properties

Hydration properties including swelling capacity (SC) and water-retention capacity (WRC) were determined.

Swelling capacity was determined by referencing the method of Mateos-Aparicio, et al. [19]. Accurately weighed pea fiber (0.3 ± 0.001 g) was added into a 25 mL graduated cylinder containing 15 mL of distilled water. The sample was stirred gently, then left undisturbed at room temperature for 8 h to completely hydrate. The volume (mL) of the settled sample was recorded, and SC was expressed as the volume of the settled sample (mL) per gram of dry fiber.

Water retention capacity was measured by referencing the method of Morales-Medina, Dong, Schalow and Drusch [11], with some modifications. Accurately weighed pea fiber (0.3 ± 0.001 g) and 15 mL of distilled water were added in a 50 mL centrifuge tube, and the sample was stirred and allowed to hydrate at room temperature for 8 h. Subsequently, the fibre suspension was centrifuged at 1790× *g* for 15 min, and the supernatant of each tube was carefully decanted. The excess of liquid was drained by turning the tubes upside down on a filter paper for several minutes. The weight of the hydrated sample (m_1_) was recorded. Then the hydrated sample was freeze-dried, and the weight of the dried sample was labeled as m_2_. WRC was calculated according to Equation (2):

(2) $$WRC\left(g/g\right)=\frac{m_{1}-m_{2}}{m_{1}}$$

### 2.9. Measurement of Oil Holding Capacity

Oil holding capacity (OHC) was measured following a modified procedure by referencing the report of Jiang, et al. [20] and Meng, et al. [21]. Pea fiber powder (0.2 ± 0.001 g) and oil (10 g ± 0.01 g) were put into a 50 mL centrifuge tube and fully mixed by vortex mixer for several minutes. Then the centrifuge tube was kept at room temperature for 4 h. Subsequently, the fibre suspension was centrifuged at 1790× *g* for 15 min, and the upper clear liquid was poured out gently. The weight of the pea fiber after absorbing oil was recorded. The OHC of pea fibers was calculated by Equation (3):

(3) $$OHC\left(g/g\right)=\frac{m_{oiled}-m}{m}$$

where m was the weight of the original ISMS-treated PeaF powder (0.2 g), and m_oiled_ was the weight of the ISMS-treated PeaF after absorbing oil (g).

### 2.10. Statistical Analysis

All experiments were carried out in triplicate using three samples, and then the mean and standard deviation were calculated by statistical analysis software (SPSS 25.0, SPSS Inc., Chicago, IL, United States). Significant differences between sample means (*p* < 0.05) were established according to Duncan’s test using one-way analysis of variance (ANOVA).

## 3. Results and Discussion

### 3.1. Particle Size Characteristics

**Figure 1:**
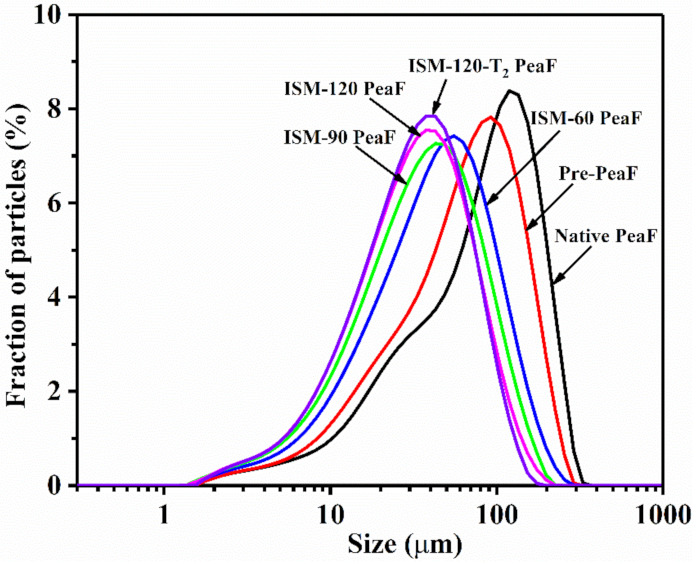
Particle size distributions of ISMS-treated PeaF.

Initially, the influence of the ISMS treatment on the particle characteristics of pea fiber was examined. Particle size distributions for native PeaF and ISMS-treated PeaF aqueous suspensions are shown in Figure 1. Native PeaF aqueous suspensions exhibited a wide and asymmetric unimodal distribution (1.4~374.8 μm) with a shoulder around 8.1~37.7 μm. The pre-pulverizer treatment weakened the shape of the shoulder and slightly narrowed the particle size distribution. After ISM treatment, peaks of the distributions tended to be homogeneously distributed. Meanwhile, a progressive shift of the peaks to the left for ISMS-treated PeaF aqueous suspensions was observed as the treatment intensity increased, and the distribution gradually narrowed. Additionally, when ISM pressure was below 120 MPa, the peaks moved downward with the increasing of ISM pressure. Although the particle size distribution of ISM-120-T_2_ PeaF was located at the leftmost, it was not significantly deviated from that of ISM-120 PeaF. These phenomena indicated that the increasing treatment pressure resulted in a decrease in the overall particle size, and ISMS treatment could grind the pea fiber to a micron size to a limited extent. As displayed in Table 1, all mean diameters analyzed decreased with increasing ISMS treatment intensity, and similar results had been found in another study of treating soybean insoluble dietary fiber by high-energy wet media milling [22]. For example, along with the treatment intensity, D_[4,3]_ values decreased from 92.6 μm of native PeaF to 38.3 μm of ISM-120-T_2_ PeaF. Furthermore, D_[3,2]_ values of pea fiber significantly dropped from 34.5 μm to 19.3 μm, and those of ISM-60 PeaF, ISM-90 PeaF, and ISM-120 PeaF were 23.5, 20.4 and 19.4 μm, respectively. Bruno, et al. [5] reported that D_[3,2]_ values of citrus fiber were approximately 26 μm and 20 μm after treatment by a M110P microfluidizer^®^ at higher pressure for one pass (103.3 MPa and 172.2 MPa, respectively). The cumulative percentiles of D_(50)_ and D_(90)_ were also reduced to varying degrees. When pressures of ISM were 90 MPa and 120 MPa, D_(90)_ the values of pea fiber were 93.2 μm and 82.5 μm, respectively. In the study of Morales-Medina, et al. [11], when D_(90)_ values of pea hull fiber reached 100 μm and 80 μm, the conditions of treatment by the LM20 Microfluidizer^®^ were predicted to be 109 MPa for two passes and 127 MPa for four passes, respectively. Meanwhile, D_(50)_ values of pea hull fiber processed by the aforementioned conditions were slightly higher than these of ISM-90 PeaF and ISM-120 PeaF in this investigation. These phenomena indicated that ISM was a more powerful technique than conventional microfluidizers at disrupting fiber to a smaller particle size. Intriguingly, ISMS treatment did not cause a decrease in span, which was contrary to the observation of narrowing distributions from Figure 1. As depicted in Figure 1, the size range of native PeaF was actually larger than that of ISMS-treated PeaF, and native PeaF were mainly large size particles with concentrated distribution. Nevertheless, ISMS-treated PeaF possessed more homogeneous distribution with a high fraction of small sized particles and a slightly small size range. The concentrated distribution of large size particles and very low fraction of small size particles contributed to a lower span for native PeaF. As considered by Guo, et al. [12], the reduced particle size of pea fiber was possibly attributed to mechanical action initiated by ISMS, and higher processing strength provided a greater crushing effect. Meanwhile, the specific surface area of pea fiber was increased with the increasing of ISMS treatment intensity, which climbed from 173.6 m^2^/kg to 309.4 m^2^/kg when ISM pressure rose to 120 MPa (Table 1). The reduction in particle size and increase in particle specific surface area initiated by the crushing effect inevitably destroyed the structure of pea fiber. Therefore, the structure of ISMS-treated PeaF will be investigated in the next sections.

### 3.2. CLSM

**Figure 2:**
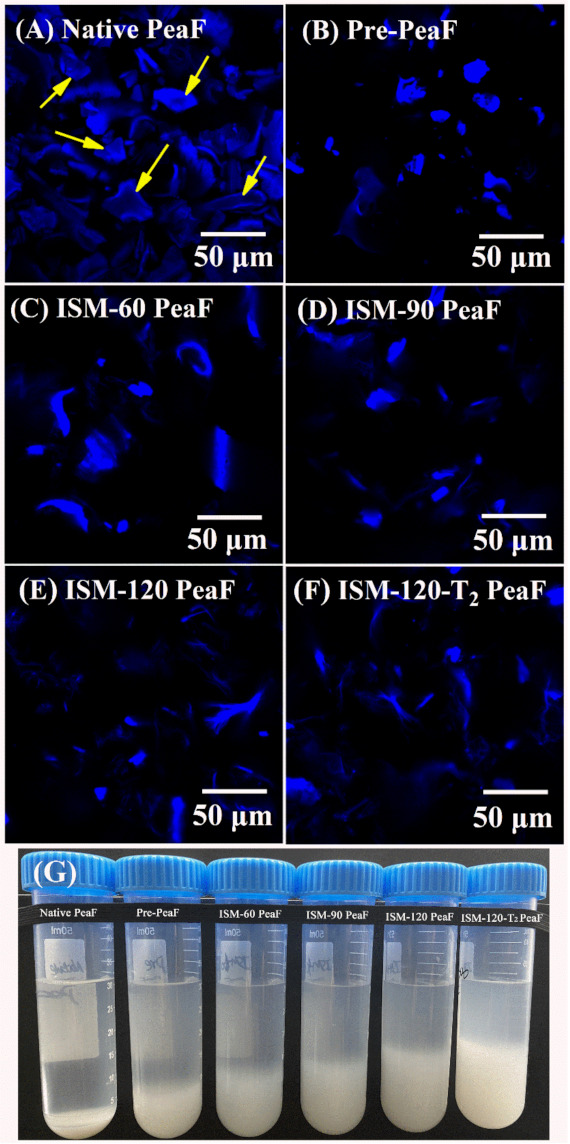
CLSM images and appearance of native PeaF and ISMS-treated PeaF. (**A**) Native PeaF; (**B**) Pre-PeaF; (**C**) ISM-60 PeaF; (**D**) ISM-90 PeaF; (**E**) ISM-120 PeaF; (**F**) ISM-120-T_2_ PeaF; (**G**) Appearance of native peaF and ISMS-treated PeaF.

The microstructure of ISMS-treated PeaF suspensions was first visualized by means of CLSM to analyze the alterations of pea fiber induced by ISMS treatment, and blue fluorescence was observed, as shown in Figure 2A–F. Native PeaF revealed the predominance of a relatively big thick and compact structure, where the centre of some fibers presented weaker fluorescence than their well-defined edges, as shown by arrows in Figure 2A. In accordance with the results of particle size, pre-pulverizer treatment broke the fibers into relatively small structures with bright fluorescence (Figure 2B). Upon ISM treatment, the dense fiber seemed to be disrupted into loose fibers with the increasing of treatment intensity according to the significant change of blue fluorescence. ISM-60 PeaF showed diverse shapes of fluorescence with some strips as depicted in Figure 2C. When ISM pressure reached 90 MPa, the fluorescence intensity of fiber was weakened. ISM-90 PeaF presented a small flake-like structure with weak fluorescence intensity (Figure 2D). The further increasing of the treatment intensity led to a greater degree of tearing fiber, and ISM-120 PeaF (Figure 2E) and ISM-120-T_2_ PeaF (Figure 2F) displayed more small fragments with faint fluorescence and multiform shape. Overall, as the intensity of the ISMS treatment increased, the fluorescence of ISMS-treated PeaF was weakened and blurred, and the area of fluorescence for most fibers significantly diminished. It was implied that the ISMS treatment disrupted the thick and compact block-like pea fibers into small loose fragments. As pictured in Figure 2G, ISMS-treated PeaF suspensions exhibited a larger sediment volume of fiber at the bottom of the centrifugal tube as the treatment intensity increased, since fibers with smaller and looser structure were more difficult to sink lower to the bottom. These results indicated that the ISMS treatment did cause serious damage to the pea fiber, which confirmed the observation of CLSM images.

### 3.3. Morphology

**Figure 3:**
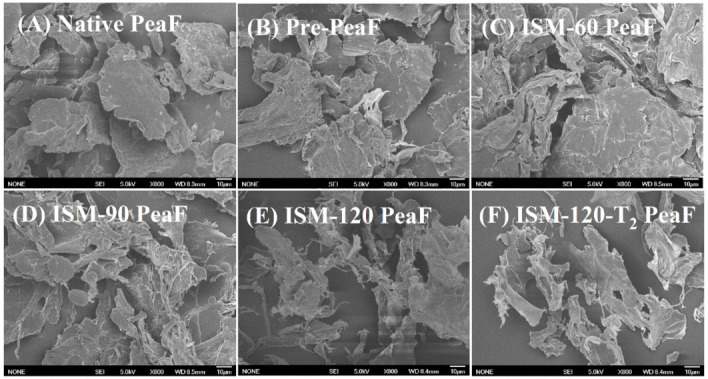
SEM micrographs. Magnification was 800×.

SEM micrographs of pea fibers before and after ISMS treatments are displayed in Figure 3. Native PeaF displayed compact and thick blocks, and some fiber blocks with a smooth surface were distributed (Figure 3A). ISMS treatment could effectively alter the microstructure of the pea fiber, and compared to native PeaF, the structure of the ISMS-treated PeaF became looser and thinner. There was a flattened structure with some band-like sheets at the edge for Pre-PeaF (Figure 3B). After ISM treatment at low pressure, the fibers were highly distorted, and ISM-60 PeaF was tightly packed into a flat plate with a large surface (Figure 3C). When the treatment pressure reached 90 MPa, the fibers were torn into flat lamellas with interwoven filaments (Figure 3D). Furthermore, the appearance of a flimsy and crimped flake-like structure was observed for ISM-120 PeaF (Figure 3E) and ISM-120-T_2_ PeaF (Figure 3F), implying that a greater degree of breaking pea fiber was induced by high treatment intensity. The change of SEM morphology was more clear than the observation of CLSM images to illustrate the effect of ISMS treatment on the pea fiber, further implying that mechanical effect initiated by ISM could destroy and break the architecture of the pea fiber. High-density energy could be produced by ISM treatment owing to powerful shear, turbulence, high-velocity impaction, high-frequency vibration, instantaneous pressure drop and cavitation forces which contributed to disrupt the pea fiber. As ISM treatment intensity increased, different degrees of damage and disruption forms of the fiber were observed. It was possible that the varied strength of mechanical forces exerted an effect on tearing the fiber when different treatment intensities were imposed. The appearance of a multi-branched flake-like structure most likely provided an explanation for the changes in the absorption properties of the fiber.

### 3.4. Bulk Density

**Figure 4:**
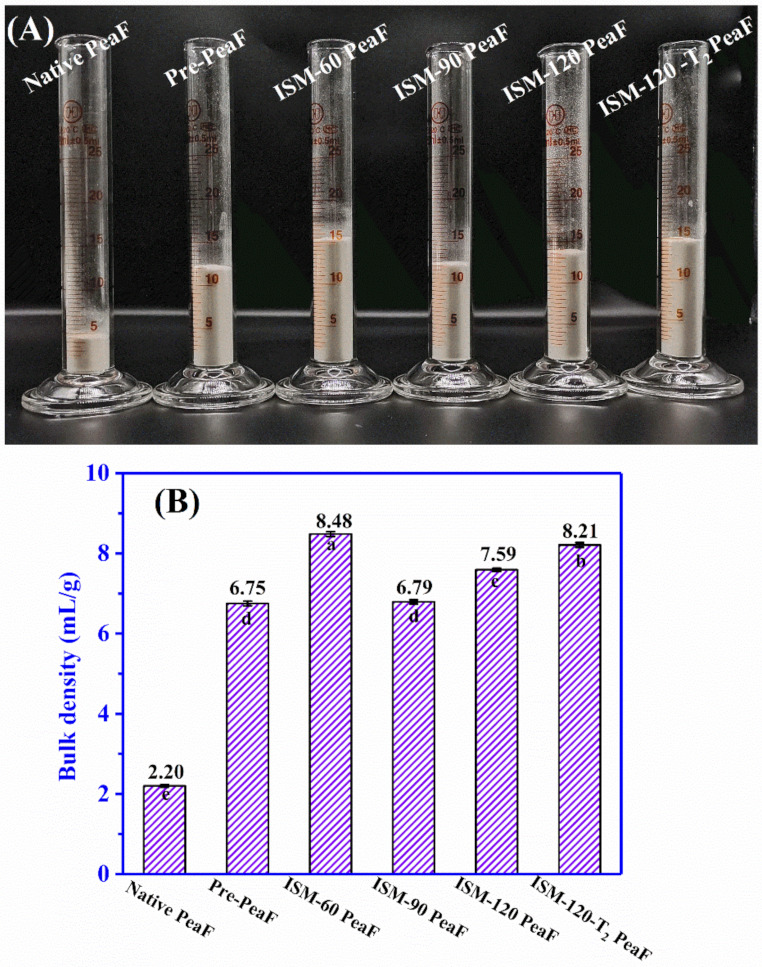
The graph of appearance volume (**A**) and bulk density (**B**) of ISMS-treated PeaF. Different letters in (**B**) indicated significant differences (*p* < 0.05) of bulk density between samples.

The visual image of the packed native PeaF and ISMS-treated PeaF powers was shown in Figure 4A. ISMS treatment observably increased the packed volume of pea fiber in varying degrees in light of the treatment intensity. Figure 4B illustrated bulk density values of fibers versus the extent of ISMS treatment. The bulk density of native PeaF was 2.20 mL/g, and pre-pulverizer treatment increased the bulk density of fiber by about 3.07 fold. While the fiber was subjected to ISM treatment at 60 MPa, the bulk density of fiber rose, reaching 8.48 mL/g. Intriguingly, in comparison with ISM-60 PeaF, applying an ISM pressure of 90 MPa resulted in a smaller increase in bulk density (6.79 mL/g). Moreover, subsequent strengthening treatment induced a continuous increase in the bulk density, which was 7.59 and 8.21 mL/g for ISM-120 PeaF and ISM-120-T_2_ PeaF, respectively. Indeed, the bulk density of fiber was related to their morphological characteristics, as mentioned by Wang, Sun, Zhou and Chen [17] and Wang, et al. [23]. It was universally acknowledged that the compact structure occupied a small space, thus native PeaF with compact and thick blocks had a low value of bulk density. As revealed in Figure 3, ISM-60 PeaF was a large dimensional flat plate in a distorted and stacked state, which produced its high bulk density. With regard to pea fibers treated by high intensity, there was more compressible space between fibers with loose flakes, thus ISM-90, ISM-120 PeaF and ISM-120-T_2_ PeaF behaved at a lower value of bulk density than the ISM-60 PeaF.

### 3.5. XRD Analysis

**Figure 5:**
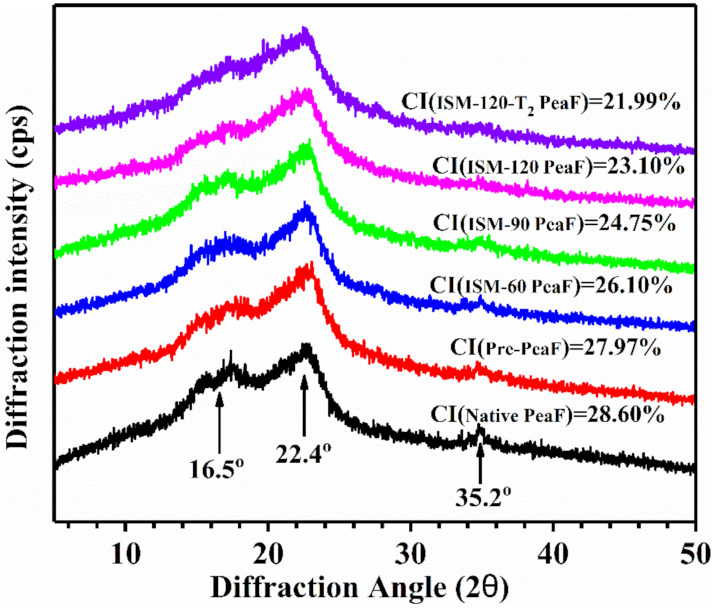
X-ray diffraction patterns of ISMS-treated PeaF.

The fiber was mainly composed of cellulose, lignin and hemicellulose, and the crystalline structure was dominated by cellulose [24]. The X-ray patterns and crystallinity of native PeaF and ISMS-treated PeaF are determined and displayed in Figure 5. There was one strong diffraction peak near the 2θ diffraction angle of 22.40° and two weak ones at about 16.5° and 35.2° in the XRD pattern of native PeaF. It was indicated that pea fiber belonged to cellulose type I, where both crystalline and amorphous regions coexist [25]. No significant difference in the peak position between native PeaF and ISMS-treated PeaF was observed. Nevertheless, the intensity of the peak near 2θ at 16.5° was visibly attenuated, and the peak near 2θ at 35.2° even disappeared with the increasing of ISMS treatment intensity, implying that the crystalline structure of pea fiber was perturbed. The crystallinity of fiber was gradually decreased from 28.60% to 21.99% along with the treatment intensity, which also suggested that the fiber was decrystallized after ISMS treatment. The weakened crystalline structure was accompanied with a reduction in the crystallinity, and this is most likely because of the destruction of the original ordered cellulose structure as analyzed by Sun, et al. [26]. Decrystallization could activate the cellulosic fiber for functionalization [18]. Therefore, the functional properties of pea fiber after ISMS treatment would be altered.

### 3.6. Hydration Properties

**Figure 6:**
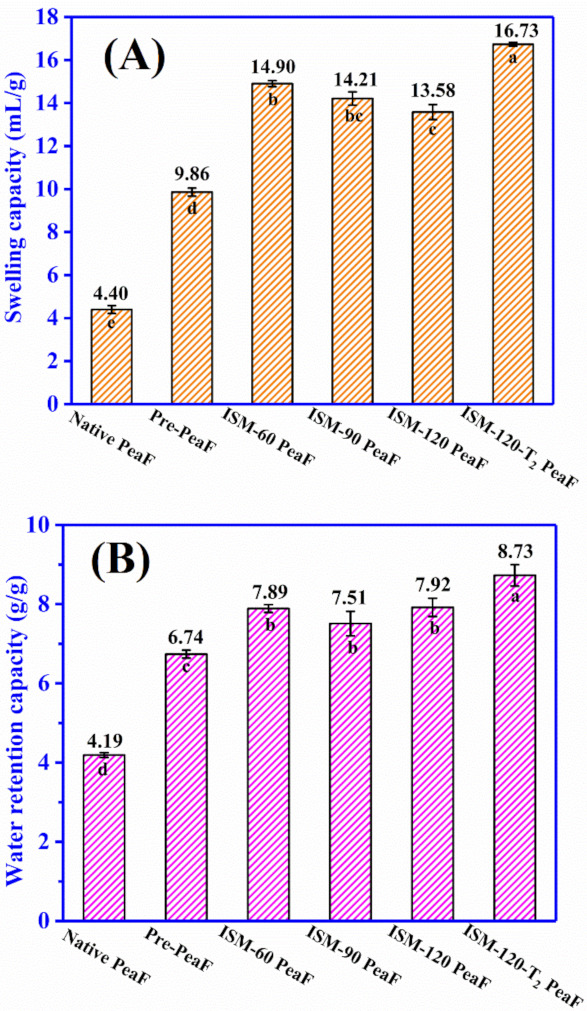
Swelling capacity (**A**) and water retention capacity (**B**) of ISMS-treated PeaF. Different letters in (**A**,**B**) indicated significant differences (*p* < 0.05) of hydration properties between samples.

The ISMS treatment-induced reduction in particle size, the increase in surface area, and changes in the microstructure and disruption of the crystalline structure could have an effect on the hydration properties of pea fiber, including swelling capacity (SC) and water retention capacity (WRC). SC and WRC of ISMS-treated PeaF is presented in Figure 6A,B, respectively. The value of SC and WRC for the native PeaF was 4.40 mL/g and 4.19 g/g, respectively. ISMS treatment significantly increased the SC and WRC of pea fiber. For instance, the SC of Pre-Pea, ISM-60 PeaF, ISM-90 PeaF, ISM-120 PeaF and ISM-120-T_2_ PeaF was 9.86, 14.90, 14.21, 13.85 and 16.73 mL/g, respectively. The change trend of WRC was similar with that of SC as the treatment intensity strengthened. In particular, ISMS treatment at 120 MPa for two passes resulted in a 3.8 fold and 2.1 fold increase of SC and WRC, respectively, and this exhibited the largest values (16.73 mL/g and 8.73 g/g). ISMS-treated PeaF occupied a larger sediment volume in Figure 2G owing to its small, loose structure, which implied a greater tendency to absorb water. The transformation from dense to loose microstructure and reduction in size owing to the mechanical effect initiated by ISMS treatment endowed pea fiber with a larger surface area and more water binding sites (polar groups etc.) to the surrounding water [27], thus leading to a strengthened expansion in water and binding with water. Deleris and Wallecan [28] also pointed out that the WRC of fiber suspensions was affected by the crystalline characteristics of cellulose, since water molecules were not able to enter into the crystalline region of the cellulose. That is, disrupting the crystalline structure by ISMS treatment also favored pea fiber to binding water. Intriguingly, the values of WHC and SC were not gradually increased with the increasing of intensity during the ISMS treatment for one pass, which was not inconsistent with other studies [29,30] in which the hydration properties of insoluble dietary fiber were increased or decreased along with the reduction in particle size. The results from this study demonstrated that the hydration properties of pea fiber were dependent on several factors, such as the alteration of the microstructure and crystallinity, and particle size did not play a vital role.

### 3.7. Oil Holding Capacity

**Figure 7:**
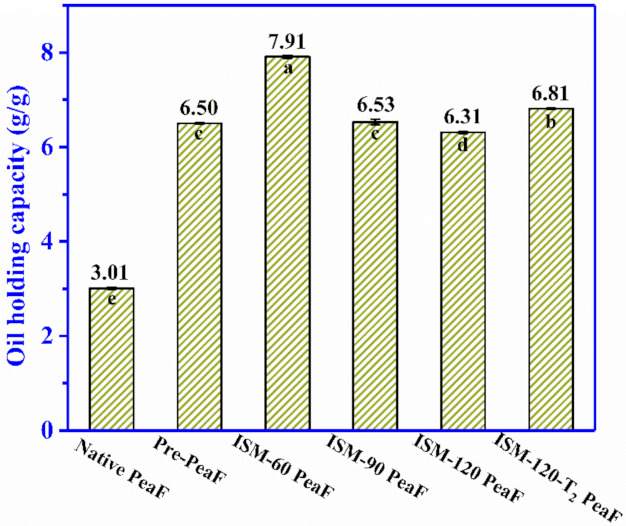
Oil holding capacity of ISMS-treated PeaF. Different letters in Figure 7 indicated significant differences (*p* < 0.05) of oil holding capacity between samples.

The intake of low-fat products using dietary fiber as a fat replacement could satisfy the requirement of lowering the amount of ingested fat and calories in the diet, so the capacity of fiber to retain oil is crucial for fibre-rich foodstuffs to exert an effect on cholesterol absorption and removing excess fat from the human body [31]. The OHC of ISMS-treated PeaF is shown in Figure 7. The OHC of native PeaF was 3.01 g/g. Similar to the effect of ISMS treatment on hydration properties, the OHC of fiber was significantly elevated. The OHC of all ISMS-treated PeaF was twice as high as that of native PeaF, and the change trend of OHC was similar to that of the bulk density with the increasing of treatment intensity. After ISMS treatment, an increase in bulk density, a more looser structure, more exposure of the fiber surface area and the disordering of the crystalline structure might increase the capillary attraction and adsorption sites of pea fibre, thus improving the oil-holding capacity. The increased OHC implied the potential of ISMS-treated PeaF to be used as an ingredient in meat products requiring oil absorption.

## 4. Conclusions

ISMS treatment significantly changed the structure of pea fiber. CLSM images revealed that fibers with a big and compact structure were disintegrated into slim and loose ones. The morphology of pea fiber was changed from compact thick blocks to flimsy crimped flakes. The crystalline structure was also destroyed, owing to the attacking of the original ordered cellulose, thus leading to the reduction of crystallinity. The alterations of the structure were accompanied with the narrowed particle size and the increased bulk density. In the meantime, the SC, WRC and OHC of pea fibers were evidently increased after the ISMS treatment. The improved hydration properties and the OHC of pea fiber was related to destroying the compact structure, providing more surface area and disrupting the crystalline structure by ISMS treatment, since more water binding and oil adsorption sites were exposed. These results suggested that the technology of ISMS facilitated the processing of pea fiber as the ingredient of foodstuffs such as meat products and jams at an industrial level. 

## Figures and Tables

**Table 1:** Particle diameter size of ISMS-treated PeaF ^1^.

Samples	Specific Surface Area (m^2^/kg)	D_[3,2]_ (μm)	D_[4,3]_ (μm)	D_(50)_ (μm)	D_(90)_ (μm)	Span
native PeaF	173.6 ± 1.7e	34.5 ± 0.4a	92.6 ± 0.5a	82.8 ± 1.2a	185.0 ± 1.4a	2.03 ± 0.05b
Pre-PeaF	197.3 ± 3.1d	30.4 ± 0.4b	72.7 ± 3.3b	64.2 ± 1.0b	145.0 ± 9.9b	2.03 ± 0.13b
ISM-60 PeaF	256.6 ± 0.1c	23.5 ± 0.1c	52.9 ± 0.4c	43.1 ± 0.1c	109.0 ± 1.4c	2.26 ± 0.03a
ISM-90 PeaF	293.4 ± 0.4b	20.4 ± 0.1d	45.0 ± 0.1d	36.1 ± 0.1d	93.2 ± 0.14d	2.31 ± 0.01a
ISM-120 PeaF	309.4 ± 1.3a	19.4 ± 0.1e	40.5 ± 0.4e	32.7 ± 0.1e	82.5 ± 1.0e	2.23 ± 0.02a
ISM-T_2_-120 PeaF	310.0 ± 2.8a	19.3 ± 0.2e	38.3 ± 0.6e	32.1 ± 0.3e	76.2 ± 1.3e	2.08 ± 0.01b

^1^ Reported results correspond to mean ± standard deviation. Different letters within the same column indicate significant differences (*p* < 0.05).

## Data Availability

Data is contained within the article.
